# IFN-gamma regulation of ICAM-1 receptors in bronchial epithelial cells: soluble ICAM–1 release inhibits human rhinovirus infection

**DOI:** 10.1186/1476-9255-5-8

**Published:** 2008-06-05

**Authors:** Suzanne C Whiteman, Monica A Spiteri

**Affiliations:** 1 School of Medicine, Keele University, Keele, UK; 2 Lung Research, Institute of Science & Technology in Medicine, Keele University, and Directorate of Respiratory Medicine, University Hospital of North Staffordshire, UK

## Abstract

**Background:**

Intercellular adhesion molecule-1 (ICAM-1) is a critical target-docking molecule on epithelial cells for 90% of human rhinovirus (HRV) serotypes. Two forms of ICAM-1 exist, membranous (mICAM-1) and soluble (sICAM-1), both expressed by bronchial epithelial cells. Interferon-gamma (IFN-γ), a crucial Th-1 immuno-regulatory mediator, can modulate mICAM-1 expression; however its simultaneous effects on mICAM-1: sICAM-1 levels and their consequent outcome on cell infectivity have not been previously explored.

**Methods:**

Primary normal human bronchial epithelial cells were pre-stimulated with IFN-γ (1 ng/ml for 24 h) and subsequently inoculated with HRV-14 or HRV-1b (TCID_50 _10 ^2.5^). Epithelial surface ICAM-1 expression and soluble ICAM-1 release were measured at the protein and gene level by immunofluorescence and ELISA respectively; mRNA levels were semi-quantified using RT-PCR. Molecular mechanisms regulating ICAM-1 isoform expression and effects on epithelial cell infectivity were explored.

**Results:**

In IFN-γ-biased cells infected with HRV-14, but not HRV-1b, mICAM-1 expression is down-regulated, with simultaneous induction of sICAM-1 release. This differential effect on HRV-14 receptor isoforms appears to be related to a combination of decreased IFN-γ-induced JAK-STAT signalling and proteolytic receptor cleavage of the membranous form in IFN-γ-biased HRV-14 infected cells. The observed changes in relative mICAM-1: sICAM-1 expression levels are associated with reduced HRV-14 viral titres.

**Conclusion:**

These findings support the hypothesis that in epithelial cells conditioned to IFN-γ and subsequently exposed to HRV-14 infection, differential modulation in the ratio of ICAM-1 receptors prevails in favour of an anti-viral milieu, appearing to limit further target cell viral attachment and propagation.

## Background

Intercellular adhesion molecule-1 (ICAM-1) is a cell surface glycoprotein, which together with its cognate ligand LFA-1 (CD18/CD11a) recruits and activates immune effector cells to sites of inflammation. Through separate domains, ICAM-1 can also serve as a crucial target-docking molecule on epithelial cells for 90% of human rhinovirus (HRV) serotypes; recognised to be involved in up to 65% of "common colds" and associated with exacerbations of chronic airways disease such as asthma and COPD. In the main, HRV respiratory infections tend to be normally brief and self-limiting in nature; the precise regulatory mechanisms for this are not fully understood. The present work explores some of the underlying processes focusing on ICAM-1 expression and regulation.

Two distinct forms of ICAM-1 receptor have been identified, membrane bound (mICAM-1) and soluble (sICAM-1) [1]; both are expressed by airway epithelial cells (EC). The present authors previously demonstrated that HRV selectively manipulates epithelial cell mICAM-1 and sICAM-1 expression in an inverse fashion to effect airway epithelial cell infection and thus promote/propagate viral respiratory episodes [2]. Membrane ICAM-1 expression can also be modulated by inflammatory cytokines, already expressed in the local milieu or induced by the viruses themselves [3-8]. Whilst the ICAM-1 gene appears to be sensitive to diverse cytokines, the magnitude and nature of each specific mediator-driven response appears to be highly dependent on cell type [9,10]. Crucially, the course of HRV infection can also be manipulated by the types and time kinetics of inflammatory cytokines in the immediate cell milieu [11-13]. Of interest, the present authors have demonstrated that IFN-γ induces down-regulation of mICAM-1 expression in HRV-14- infected cells with consequent decrease in viral titres and cell infection [14]. As this suppressor action contrasts with induction of same cell receptor by IFN-γ in the *absence *of infection, it is unclear whether IFN-γ-induced decrease in infected cell mICAM-1 levels can solely explain consequence on HRV infectivity. Indeed the effect of IFN-γ on soluble ICAM-1 isoforms during HRV infection has not been described to date. This is of interest as IFN-γ is a key anti-viral lymphokine in the host immune response against viral infections. The present authors hypothesise that IFN-γ biased cells exposed to HRV-14 may selectively modulate own ICAM-1 receptor levels, promoting a down-regulation of mICAM-1 expression, whilst inducing sICAM-1 release into the local milieu. A shift towards the sICAM-1 variant would favour abrogation or limitation of HRV infection, and may explain, albeit partly, the self-limiting nature of rhinovirus infections observed *in vivo*. In support, sICAM-1 has been shown to possess anti-viral properties both *in vitro *[15] and *in vivo *[16,17].

To test this hypothesis, in this current study a series of experiments were designed first, (a) to determine the influence of IFN-γ alone on bronchial epithelial cell expression of both mICAM-1 and sICAM-1 forms at the protein and gene level; and (b) to examine the consequences of IFN-γ cell preconditioning on subsequent HRV binding and infection. To ensure that the observed effects of the major group rhinovirus, HRV-14, on ICAM-1 expression are receptor mediated, a minor group rhinovirus (HRV-1b) which utilises a separate low density lipoprotein receptor, and not ICAM-1, was used as control. As IFN-γ is known to act via a STAT-1 dependent pathway [18], the present work next explored the role of this signal transducer in the regulation of mICAM-1 during HRV infection. Furthermore, potential pathways of sICAM-1 production, including proteolytic cleavage, were explored using inhibitors of gene transcription and protein synthesis as well as protease inhibitors.

## Methods

### Epithelial cell cultures and viral stocks

Commercially available normal human bronchial epithelial cells (NHBE) (three separate sources) were obtained from Clonetics Corporation Walkersville MD USA. NHBE cells were cultured in small airway basal medium (SABM) supplemented with epidermal growth factor (25 ng/ml), hydrocortisone (0.5 μg/ml), insulin (5 μg/ml), transferrin (10 μg/ml), epinephrine (0.5 μg/ml), triiodothyronine (6.5 ng/ml), bovine pituitary extract (52 μg/ml), retinoic acid (0.1 ng/ml), gentamicin (50 mg/ml), amphotericin B (50 μg/ml) at 37°C in humidified 5% CO_2_/air. All reagents were supplied by Clonetics (above). In subsequent experiments, NHBE cells were seeded in 25 cm^2 ^flasks at a density of 125 000 cells/flask and utilised when 70–80% confluent.

The major group rhinovirus seed (HRV-14) was kindly donated by J. Kent (University of Leicester UK) and the minor group rhinovirus (HRV-1b) was donated by S. Johnston (Imperial College, London). A stock solution of both HRV-14 and HRV-1b rhinoviruses were generated by infecting confluent monolayers of HeLa Ohio cell line as described previously [14]. Briefly, confluent monolayers of Hela cells were inoculated with either HRV-14 or HRV-1b at a known dilution (10^2.5^TCID_50_/ml) and incubated for 90 mins at 34°C in humidified 5% CO_2_/air. After which, cells were cultured until cytopathic effect (CPE) was > 80%. Medium containing virus was centrifuged at 600 g for 10 mins; then viral suspension was stored at -80°C until use.

Prior to use, viral stocks (HRV-14 and HRV-1b) were purified using a sucrose gradient. 20 μg/ml RNase A (Sigma, UK) was added to the viral suspension and incubated at 35°C for 20 mins. 1% sodium sarkosyl (Sigma-Aldrich, UK) and 2-mercaptoethanol (1 μg/ml) were added to the RNase treated viral suspension. This was then overlayed on 1 ml of purification solution (20 mM Tris Acetate, 1 M NaCl, 30% w/v sucrose) and centrifuged at 200 000 g for 5 h at 16°C. The supernatant was discarded and the resulting virus pellet was resuspended in medium and stored at -80°C until required.

### IFN-γ treatment of NHBE cells

To determine effect of the Th-1 cytokine, IFN-γ, on expression of ICAM-1 isoforms in airway epithelial cells, medium was removed from cell cultures containing 70–80% confluency and replaced with either media containing IFN-γ (1 ng/ml, R & D Systems, Abingdon UK) or standard SABM media (controls) for 24 h at 37°C in humidified 5% CO_2_/air. Prior studies had demonstrated stated concentration and time period as optimum for maximal induction of in mICAM-1 expression (14). All experiments were set up in triplicate and repeated for each donor NHBE culture. Media containing IFN-γ and standard SAGM media were then removed and cell monolayers washed with PBS.

### HRV infection of NHBE cells

To determine whether expression behaviour of ICAM-1 isoforms in HRV-infected airway epithelial cells is influenced by IFN-γ pre-conditioning (i.e. IFN-γ-biased target cells), parallel cultures of untreated and IFN-γ-treated NHBE cell monolayers were exposed to SABM media containing HRV-14 (10^2.5^TCID_50_/ml, previously determined as the optimum dose, see ref 14), HRV-1b (10^2.5^TCID_50_/ml) or virus free media for 90 mins at 34°C, 5% CO_2_/air. The cells were then washed 3 times in PBS and standard SABM medium was replaced on all cell monolayers to sustain cell growth. The gene and protein expressions of membrane and soluble ICAM-1 forms were measured simultaneously at 0, 8, 24 and 96 h post infection. 0 h represents the point immediately after 90 min inoculation period; subsequent time points (8 to 96 h) are taken from this 0 h. of viral infection. ICAM-1 is the cellular receptor for the major group of human rhinoviruses, HRV-14; as control, another viral strain (minor group rhinovirus HRV-1b, which utilises a separate low density lipoprotein receptor) was included to determine whether observations were receptor mediated. All experiments were set up in triplicate and repeated for each donor NHBE culture. In all cultures, cells and supernatants were recovered at 0 h, 8 h, 24 h, and 96 h. for further evaluation; supernatants were stored at -70°C for soluble ICAM-1 level assays and viral titre analysis.

### sICAM-1 protein ELISA

Soluble ICAM-1 assays were performed with a commercially available ELISA kit (BioSource International, California USA). The minimum detectable level of human soluble ICAM-1 (hsICAM-1) was < 0.04 ng/ml. 100 μl of undiluted cell culture supernatant or standard were utilised in the assay, which was performed in accordance with the manufacturer's guidelines.

### Immunofluoresecence analysis of mICAM-1 protein

Membrane bound ICAM-1 expression and localisation was evaluated at 0 h, 8 h, 24 h, and 96 h post HRV infection. At each time point, the cells were collected via trypsinisation and centrifugation; 6 cytospins for each experimental condition at each time point were prepared for immunofluorescence staining; remaining cells were utilised for RNA extraction, cDNA synthesis and RT-PCR. Internal controls consisted of unstimulated and uninfected cells at each time point to allow comparisons between controls and IFN-γ treated/infected cells. A cytokeratin immunoglobulin IgG-specific monoclonal antibody (Sigma-Aldrich, UK) was used to confirm the epithelial origin of NHBE cell lines used. NHBE cells were incubated with ICAM-1 monoclonal antibody at a concentration of 5 μg/ml, (R1/1.1, IgG Boehringer Ingelheim, USA) at room temperature for 30 mins. After washing thoroughly with PBS, a secondary anti-mouse IgG FITC conjugated antibody (Sigma Aldrich, Dorset, UK) used at 1:500 dilution for 30 minutes at room temperature. The cells were then washed and mounted using Vectashield^® ^mounting medium containing DAPI (Vector laboratories, Peterborough, UK). Slides were viewed under epiflourescence with filter set at 450–490 nm for FITC and 340–380 nm for DAPI. Images were obtained using a Leica DC200 digital camera and software (Leica Microsystems, Heerbrugg, Switzerland). For each experimental condition, 500 cells from five random fields of view were imaged and analysed for each experimental condition; all slides were coded prior to analysis and read blind. The data is expressed as the percentage of positive stained cells ± S.E.M.

### RNA extraction and reverse transcription

Total RNA was extracted from NHBE cells at 0, 8, 24 and 96 h post infection using Trizol (GibcoBRL, Paisley UK) according to the manufacturer's guidelines; cDNA was synthesised from 2 μg of RNA. cDNA synthesis was conducted in a reaction mixture containing 20 pmol oligo dt primer, 5× buffer (50 mM, pH 8.3 75 mM KCl 3 mM MgCl_2_), 0.5 mM dNTP mix, 0.5 units RNase inhibitors and 200 units MMLV reverse transcriptase; the total reaction volume was 20 μl. All cDNA synthesis reagents were obtained from Clontech (Palo Ato USA). This mixture was then incubated at 42°C for 1 hr, after which the reverse transcriptase and DNase were heat inactivated at 94°C for 5 mins. The cDNA was diluted to a final volume of 100 μl and stored at -80°C for Reverse Transcription Polymerase chain reaction (RT-PCR).

### Detection of m and sICAM-1 gene expression using RT-PCR

Glyceraldehyde-3-phosphate dehydrogenase (G3PDH) was used as a control for cDNA synthesis and RT-PCR. Primers used to detect G3PDH were 5' TGA AGG TCG GAG TCA GA 3' (sense) and CAT GTG GGC CAT GAG GTC CAC CAC (antisense). Primers for the detection of mICAM-1 and sICAM-1 were based on Wakatsuki *et al *(1995) [19]. The sequence of the sense primers used to detect mICAM-1 and sICAM-1 was 5'CAA GGG GAG GTC ACC CGC GAG GTG 3' and 5' CAA GGG AGG TCA CCC GCG AGC C 3' respectively. Both primers were used in combination with a common antisense primer with the following sequence 5' TGC AGT GCC CAT TAT GAC TG 3'. The RT-PCR consisted of 25 pmol primers, 200 μM dNTPs, 1.5 mM MgCl_2_, 5 μl 10× PCR buffer and 2.5 U Amplitaq Gold (Perkin-Elmer, Warrington UK) in a 50 μl reaction mixture in a thermal cycler (PTC 200 Pielter Thermal cycler) under the following conditions:- 95°C for 12 mins, 94°C for 1 min and 15 secs (denaturation step), 60°C (G3PDH) or 65°C (ICAM-1) for 1 min and 15 secs (annealing step) and 72°C for 1 min (extension step) for a total of 30 cycles (G3PDH) or 35 cycles (ICAM-1), after which a final extension step was performed at 72°C for 10 mins. RT-PCR products were resolved using 3% metaphor agarose (Flowgen, Litchfield UK) gel in TBE buffer (89 mM Tris, 89 mM boric acid, 2 mM EDTA, Sigma-Aldrich UK). Gels were visualised using Ethidium Bromide and UV light, and analysed densitometrically (Model GS-670, BioRad, Hemel Hempstead, UK) using Molecular Analyst (version 1.5) (BioRad, UK). Restriction endonucleases were used to confirm the size of the RT-PCR products. Membrane bound ICAM-1 RT-PCR products were digested to give product sizes of 45 base pairs and 57 base pairs and soluble ICAM-1 RT-PCR products were digested at the site of the deletion to give 63 bp and 20 bp products. In addition, to confirm the presence of the 19 bp deletion, RT-PCR amplicons were sequenced using a ABI PRISM automated sequencer model 310 (Perkin Elmer, California, USA).

### Regulation of ICAM-1 receptors

To enquire at which level, and how, IFN-γ may regulate expression and release of ICAM-1 isoforms in uninfected and HRV-infected NHBE cells, a series of different approaches was adopted. In a first set of experiments two different pharmacological inhibitors were used.

#### Inhibition of de novo protein synthesis

IFN-γ-naive NHBE cell cultures were first pre-incubated for 2 h at 37°C, humidified 5% CO_2_/air with 10 μg/ml of cycloheximide (Sigma-Aldrich, Dorset); used for its known inhibition of protein synthesis [20]. Cells were then washed and incubated with media containing IFN-γ (1 ng/ml) or standard SABM for 24 h; after which some cultures were infected with HRV-14 as described above. Levels of membranous and soluble ICAM-1 levels were then determined as above.

#### Inhibition of gene transcription

In others cultures, actinomycin D (Sigma-Aldrich, Dorset), an inhibitor of gene transcription was used to block the effects of IFN-γ and HRV-14 on ICAM-1 gene transcription. NHBE cells were incubated with actinomycin D at a concentration of 10 μg/ml (identified as optimum dose in previous dose- response experiments; data not shown) for 2 h at 37°C in humidified 5% CO_2_/air. Cell monolayers were washed and incubated with IFN-γ conditioned media (1 ng/ml) or standard SABM media. Appropriate wells were infected with HRV-14 as above. Levels of membranous and soluble ICAM-1 levels were then determined as above.

#### Role of JAK/STAT-1 pathway in regulation of mICAM-1 expression

In a second set of experiments, knowing that IFN-γ could mediate its effects via the JAK/STAT signalling pathway, total and phosphorylated forms of STAT-1 were evaluated using Western blotting. NHBE cells were cultured in SABM and incubated with IFN-γ (1 ng/ml) for 24 h; followed by infection with HRV-14 (TCID_50 _10^2.5^) for specified short time points (0 min, 5 min, 10 min and 30 min) to capture early initiation transcription events. NHBE cells were then lysed at these short time points using a buffer containing 1% Triton-X100, 20 mM Tris HCL (pH 8.0), 137 mM sodium chloride (NaCl), 10% glycerol, 1 mM sodium orthovanadate, 2 mM EDTA, 1 mM phenylmethylsulfonyl fluoride (PMSF), 20 μM leupeptin and 0.15 U/ml aprotinin. All reagents were molecular biology grade (Sigma-Aldrich, UK). The cells were placed on ice for 20 min; total protein was collected by centrifugation and assayed using a commercially available kit based on the Lowry assay (Biorad, UK). 25 μg reduced protein samples were electrophoresed on 12.5% SDS-PAGE and transferred to nitrocellulose membranes sandwiches (0.45 μm pore size Novex, San Diego USA). Molecular weight markers were run with the samples. Membranes were blocked with 10% w/v low-fat milk for 1 h in TBS-T and probed for 2 h with mouse anti-human anti-STAT-1 (Upstate Technology UK) diluted 1:1000 in TBS-T or mouse anti-human anti-phosphoSTAT-1 (generous gift from R. A. Knight, Imperial College, London). Membranes were incubated with a horseradish peroxidase conjugated rabbit anti-mouse antibody (Dako, Denmark) diluted 1:20,000 in TBS-T for 1 h. The ECL system was used for detection (Amersham, Buckinghamshire, UK). The membranes were re-probed with a control mouse IgG as a negative control, and vinculin (Lab Vision, USA) as a control for sample loading. The membranes were analysed densitometrically (Model GS-670, BioRad, UK) using Molecular Analyst Software (version 1.5) (BioRad, UK) and normalised against vinculin and expressed as a ratio to total STAT-1.

To further support a key role for the JAK/STAT-1 pathway in the regulation of ICAM-1 expression, the present authors performed separate experiments incorporating different concentrations of AG490 (Sigma-Aldrich, UK) to inhibit JAK1/2. These experiments focused on examining the consequences of inhibiting JAK1/2 on ICAM-1 expression at the gene and protein level. To achieve this NHBE cells were incubated with AG490 overnight and ICAM-1 expression analysed at 24 h post HRV-14 infection to capture the effects of AG490 on both m and s ICAM-1 expression. NHBE cells were incubated with AG490 at 20 μM, 40 μM, and 80 μM overnight at 37°C in humidified 5% CO_2_/air; the cell monolayers were then washed and incubated with IFN-γ conditioned media (1 ng/ml) or standard SABM media for 24 h at 37°C in humidified 5% CO_2_/air. After which, both the unconditioned and IFN-γ conditioned media were removed; cell monolayers washed 3 times in PBS and then infected with SABM media containing HRV-14 (10^2.5^TCID_50_/ml) or virus free media for 90 mins at 34°C, 5% CO_2_/air. At 24 h post HRV infection cell monolayers were washed 3 times in PBS and cells were collected via trypsinisation and centrifugation for miCAM-1 protein analysis. 6 cytospins for each experimental condition were prepared for immunofluorescence performed as described above. For each experimental condition, 500 cells from five random fields of view were imaged and analysed for each experimental condition. The data is expressed as the percentage of positive stained cells ± S.E.M.

#### Inhibition of proteolytic cleavage of mICAM-1

As the down-regulation of membranous ICAM-1 was accompanied by a simultaneous increase in the release of sICAM-1 in response to both IFN-γ stimulation and infection with HRV-14, the present authors hypothesised that proteolytic cleavage of mICAM-1 may, albeit partly, be responsible for these observations. To test this hypothesis, NHBE cells were treated with a broad spectrum protease inhibitor cocktail containing serine, cysteine, metalloprotease and calpain inhibitors (Complete™ Boehringer Ingelheim, Germany) at a concentration of 1 Complete™ mini tablet/10 ml media overnight. NHBE cells were then stimulated with IFN-γ (1 ng/ml for 24 h) and infected with HRV-14 (10^2.5^TCID_50_/ml for 90 min). Levels of membranous and soluble ICAM-1 levels were then determined at 0 h, 8 h, 24 h, and 96 h as described above.

### Viral titre assay

The TCID_50 _method was used to calculate the concentration of the virus in cell culture supernatants at 0, 8, 24, 96, 120 and 144 h. Serial dilutions of cell culture supernatants were incubated in cell monolayers in 96 well plates for 5 days at 34°C in humidified air containing 5%CO_2_. The presence of cytopathic effect (CPE) in the wells was used to calculate the TCID_50 _using the Karber formula [13-15].

### Statistical Methods

Means ± standard error (SE) were calculated, and statistically significant differences between experimental conditions and controls were determined by repeated measure Anova test at p < 0.05. We utilised the Tukey-Kramer HSD test to perform *post hoc *comparisons between the mean values of different epithelial cell treatments.

## Results

### Down-regulation of mICAM-1 expression: response to IFN-γ and HRV infection

**Figure 1:**
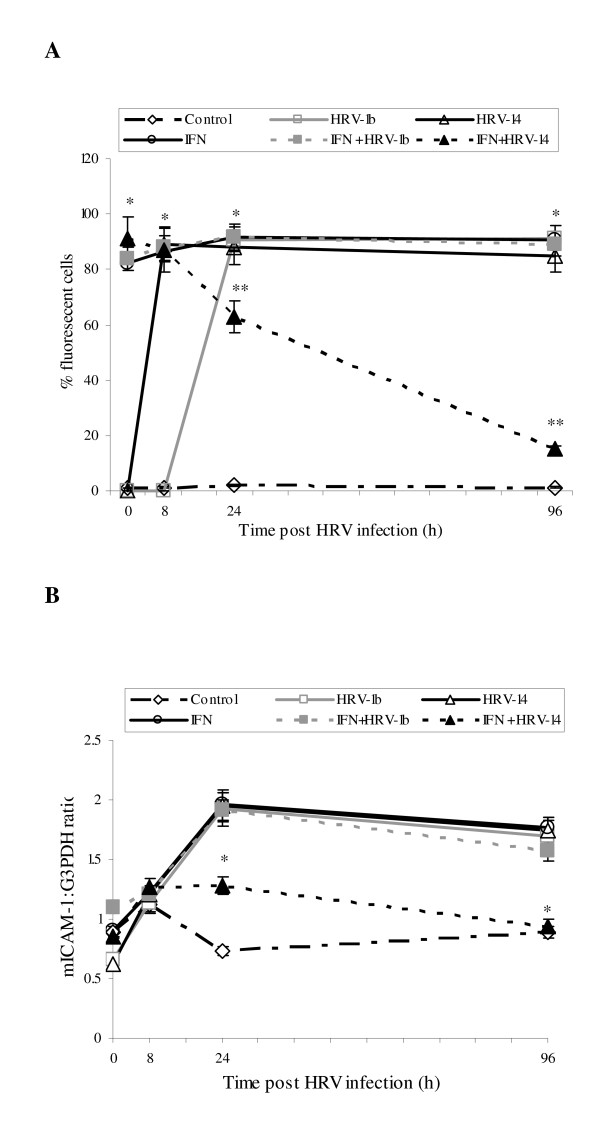
**Effect of IFN-γ cell preconditioning (1 ng/ml for 24 h) singly and with HRV-14 or HRV-1b infection (TCID_50 _10^2.5 ^for 90 mins) on mICAM-1 protein expression (A) and mICAM-1 gene expression (B) in NHBE cells over the study period (0–96 h; 0 h representing the point immediately after 90 min inoculation period)**. mICAM-1 protein expression was measured using immunofluorescence. Data are mean ± S.E. of three separate experiments (Fig. 1A, *p < 0.05, IFN-γ treated uninfected and infected cells compared to control untreated and uninfected cells, **p = 0.02, IFN-γ treated HRV-14 infected cells compared to IFN-γ treated uninfected cells and IFN-γ treated HRV-1b infected cells). Gene expression of mICAM-1 was semi-quantified using RT-PCR and densitometry and expressed as a ratio to the housekeeping gene, G3PDH. Data are mean ± S.E. of three separate experiments (Fig. 1B, *p < 0.05, IFN-γ treated infected cells compared to IFN-γ treated uninfected cells and IFN-γ treated HRV-1b infected cells).

The present authors describe first the effects of the Th-1 mediator IFN-γ on expression behaviour of mICAM-1 isoform in NHBE cells, and how expression was altered in the presence of HRV infection. Observed patterns of expression were consistent across the different source NHBE cultures. mICAM-1 protein did not change in control untreated, uninfected cells throughout the test 96 h culture period (Fig. 1A). HRV infection alone induced significantly mICAM-1 expression, peaking at 8 h and 24 h with HRV-14 and HRV-1b respectively (**p = 0.02) compared to control untreated uninfected cells), and remained elevated to the end of the culture period. Cell-preconditioning with IFN-γ (1 ng/ml) in both uninfected and infected cells also enhanced mICAM-1 expression at 0 and 8 h (*p < 0.05 compared to untreated uninfected cells). Upregulation of mICAM-1 was sustained in IFN-γ treated uninfected cells and IFN-γ treated cells infected with HRV-1b (minor group rhinovirus) over 96 h. However, in IFN-γ treated HRV-14-infected equivalent cells, levels of mICAM-1 progressively fell to basal levels by 96 h (*p < 0.02 compared to IFN-γ treated uninfected cells and IFN-γ treated HRV-1b infected cells). Trypan blue exclusion assays demonstrated that IFN-γ and HRV infection had no significant effect on cell viability (data not shown).

A similar pattern of expression was observed at the level of mRNA. Increased mICAM-1 mRNA levels were observed in IFN-γ pretreated uninfected cells and IFN-γ-naive HRV-1b and HRV-14 infected at 24 and 96 h (Fig. 1B, *p = 0.01) compared to untreated uninfected cells). Levels of mICAM-1 mRNA were also up-regulated in IFN-γ treated HRV-1b infected cells (*p < 0.05, compared to untreated uninfected cells). In contrast, mICAM-1 mRNA levels in IFN-γ treated HRV-14 infected cells were significantly down-regulated at 24 h (*p < 0.05 compared to IFN-γ treated uninfected cells, untreated HRV-14 infected cells and untreated HRV-1b), returning to near-basal levels by 96 h (*p < 0.05 compared to IFN-γ treated uninfected cells, untreated HRV-14 infected cells and untreated HRV-1b).

Minor group HRV-1b was used in control experiments to examine whether the observed reduction in mICAM-1 in response to IFN-γ and HRV-14 was receptor restricted as HRV-1b is known to utilise a different receptor. Even though HRV-1b induced mICAM-1 expression, it did not have the same down-regulatory effect on mICAM-1 in the presence of IFN-γ as HRV-14. Therefore subsequent experiments focussed on the investigation of the molecular mechanisms responsible for differential regulation of ICAM-1 receptors in response to IFN-γ in response to major group HRV infection only.

### Inhibition of de novo protein synthesis and gene transcription

**Figure 2:**
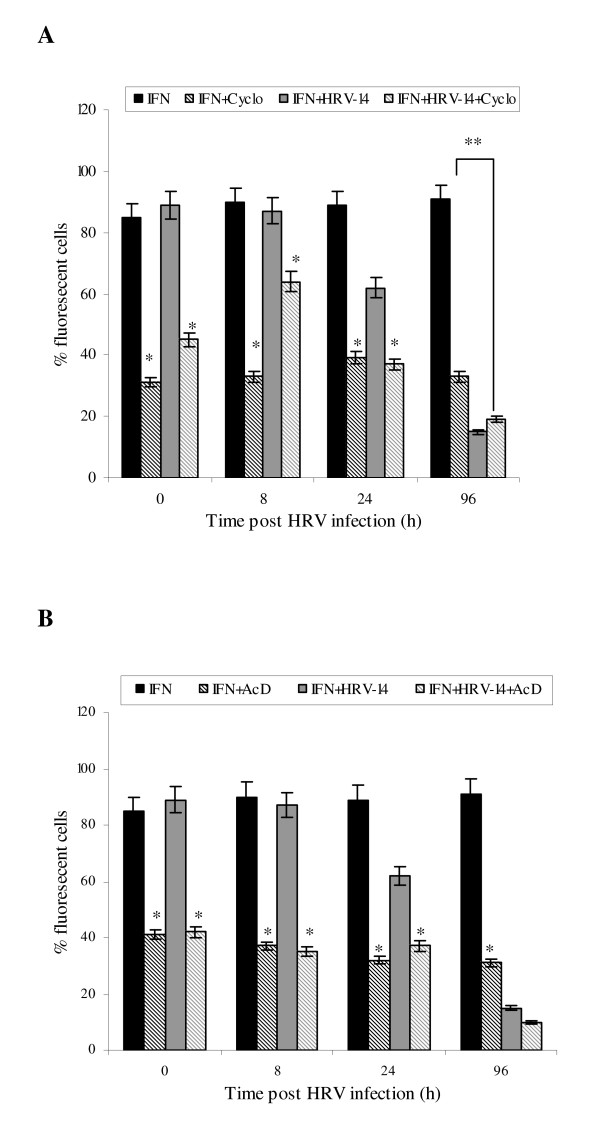
**Effect of cycloheximide (fig 2A, 10 μg/ml for 2 h) and actinomycin D (Fig 2B, 10 μg/ml for 2 h) on mICAM-1 surface expression in IFN-γ treated uninfected and HRV-14 infected cells**. Data are mean ± S.E. of three separate experiments (Fig. 2A, *p < 0.05, cycloheximide treated cells compared with equivalent un-treated cells, **p < 0.05, IFN-γ treated uninfected cells compared with IFN-γ treated infected cells, Fig. 2B, *p < 0.05, actinomycin D treated cells compared with equivalent untreated cells).

To examine at which level IFN-γ regulated expression of ICAM-1 in uninfected and HRV-14 infected NHBE cells, we first used two different pharmacological inhibitors, cycloheximide and actinomycin D to block *de novo *protein synthesis and gene transcription respectively. Cycloheximide treatment (10 μg/ml) of NHBE cells induced a down-regulation of mICAM-1 protein expression irrespective of the presence of IFN-γ and HRV infection up to 24 h (*p < 0.05, Fig 2A). This reduced expression continued up to 96 h in uninfected cells (*p < 0.05, Fig 2A). Similar pattern effects on mICAM-1 gene transcription were observed in equivalent NHBE cell cultures pretreated with actinomycin D (10 μg/ml) (Fig 2B). The present authors postulate that the failure of cycloheximde and actinomycin D to cause a complete abrogation of mICAM-1 expression may be due to co-existence of other mechanisms mobilising existent intracellular ICAM-1 stores.

### STAT-1 activation is decreased in HRV-infected cells

**Figure 3:**
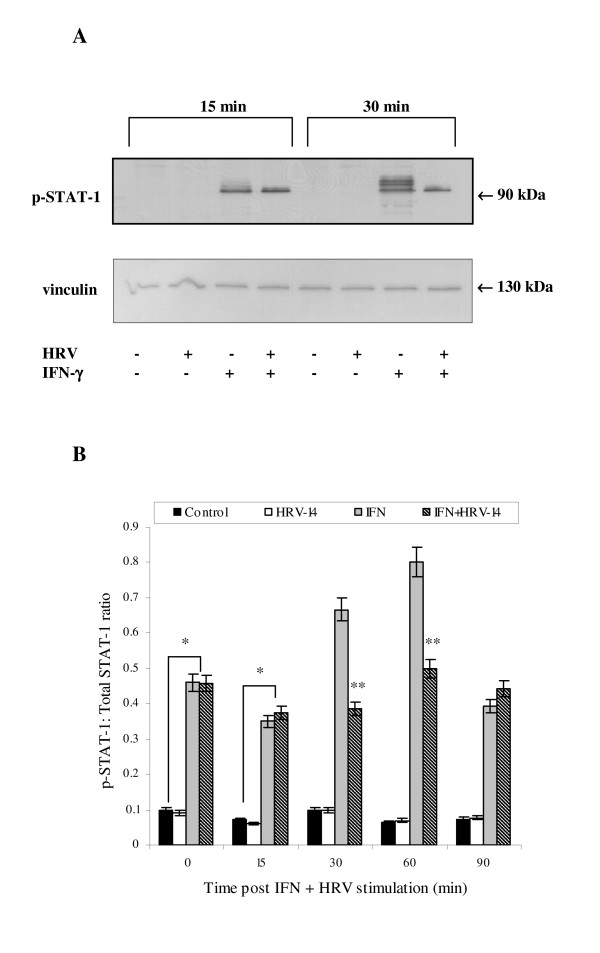
**Effect of HRV-14 on STAT-1 levels in NHBE cells at 0–90 min of infection with HRV-14 (TCID_50 _10^2.5^)**. Phosphorylated STAT-1 was analysed using Western blotting. Fig. 3A is a representative blot illustrating phosphorylated STAT-1 (90 KDa) levels at 15 and 30 min of infection. Vinculin was analysed using Western blotting as a control for sample loading. Western blots from three separate experiments were analysed densitometrically (Fig. 3B) normalised against vinculin and expressed as a ratio to total STAT-1. Data are mean ± S.E. (*p < 0.05 compared to IFN-γ treated uninfected cells at equivalent time points and **p < 0.04 IFN-γ treated uninfected cells compared to control untreated and uninfected cells).

**Figure 4:**
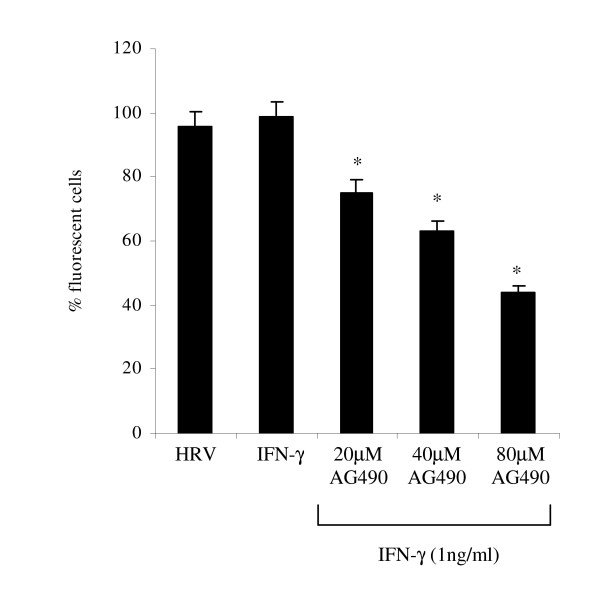
**Effect of AG490 (20–80 μM) on mICAM-1 protein levels in IFN-γ treated uninfected cells**. mICAM-1 expression was evaluated using immunofluoresence. Data are mean ± S.E. of three separate experiments (Fig. 4A, *p < 0.05, Cells treated with AG490 prior to stimulation with IFN-γ compared to cells stimulated with IFN-γ alone).

Following on above findings, the authors sought to explore whether observed effects in IFN-γ-biased NHBE cells on mICAM-1 levels during HRV-14 infection could be mediated via a JAK/STAT signalling pathway; STAT-1 being a well-known signal transducer activated by IFN-γ. As expected, in uninfected cells, IFN-γ induced a strong band corresponding to phosphorylated STAT 1, approximately 90 KDa in size at all experimental time points (0–90 mins) (Fig. 3A and 3B). However, in HRV-14 infected cells, IFN-γ induction of phosphorylated STAT-1 was weaker at 30 and 60 mins (Fig. 3B, **p < 0.04), which appeared to correspond to the previously observed reduction in mICAM-1 expression (Fig 1). Next, to confirm the important function of JAK/STAT-1 signalling in the induction of mICAM-1, uninfected and HRV-14 infected cells were pre-treated with a range of concentrations of the JAK2 inhibitor, AG490. As shown in Fig 4, IFN-γ mediated induction of mICAM-1 levels was inhibited in the presence of AG490 in uninfected cells, at all three concentrations used. However the authors failed to observe similar inhibition with AG490 in HRV-infected cells (data not shown). AG490 had no significant effect on epithelial cell viability as assessed with trypan blue exclusion (cell viability 97 ± 2.2%).

### Increase in sICAM-1 release by IFN-γ and HRV-14 infection

**Figure 5:**
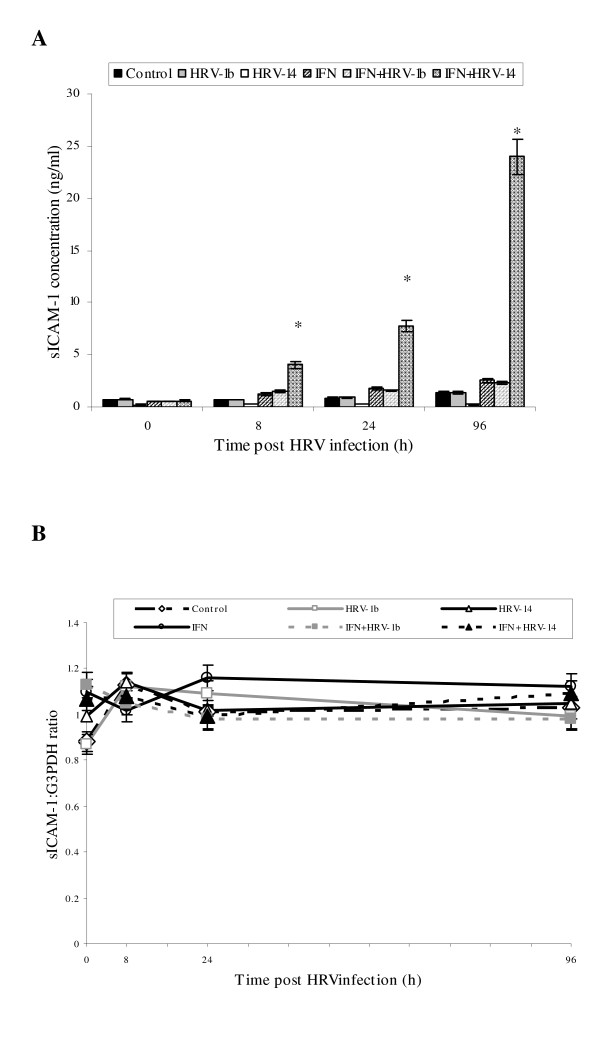
**Effect of IFN-γ cell preconditioning (1 ng/ml for 24 h) singly and with HRV-14 or HRV-1b infection (TCID_50 _10^2.5 ^for 90 mins) on sICAM-1 protein release (A) and sICAM-1 gene expression (B) in NHBE cells over the study period**. sICAM-1 in cell culture supernatants was assayed using ELISA. Data are mean ± S.E. of three separate experiments (Fig 5A,*p < 0.05, IFN-γ treated HRV-14 infected cells compared to IFN-γ treated uninfected cells and IFN-γ treated HRV-1b infected cells, **p = 0.02, IFN-γ treated infected cells compared to IFN-γ treated uninfected cells and IFN-γ treated HRV-1b infected cells). Gene expression of sICAM-1 was semi-quantified using RT-PCR and densitometry and expressed as a ratio to the housekeeping gene, G3PDH. Data are mean ± S.E. of three separate experiments

In this present study, the simultaneous effect of IFN-γ on the soluble ICAM receptor was also explored to determine patterns of mICAM: sICAM-1 isoform differential processing in uninfected and infected NHBE cells under same test conditions. As previously reported by the authors [2], sICAM-1 protein was undetected in untreated HRV-14 infected cell cultures at any time point. IFN-γ induced a small but significant increase in sICAM-1 release in uninfected cells and cells infected with the minor group rhinovirus, HRV-1b (Fig. 5A *p < 0.05 IFN-γ treated uninfected and IFN-γ treated HRV-1b infected cells versus control untreated uninfected cells). In contrast, when equivalent IFN-γ treated cells were infected with HRV-14, sICAM-1 release is up-regulated in a time- dependent fashion from 0 to 96 h (*p < 0.05, Fig. 5A), with levels reaching a 20-fold increase over IFN-γ stimulated uninfected NHBE cells and controls (**p < 0.03, Fig. 5A) at 96 h.

**Figure 6:**
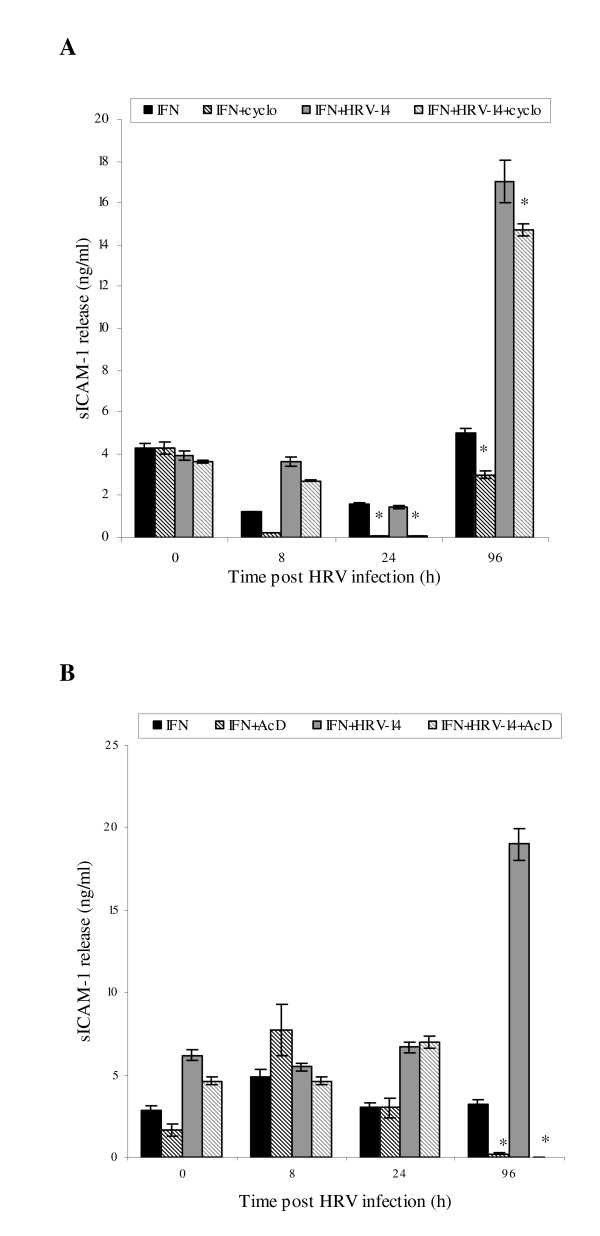
**Effect of cycloheximide (fig 6A, 10 μg/ml for 2 h) and actinomycin D (Fig 6B, 10 μg/ml for 2 h) on sICAM-1 expression in IFN-γ treated uninfected and HRV-14 infected cells**. Data are mean ± S.E. of three separate experiments (Fig. 6A, *p < 0.05, cycloheximide treated cells compared with equivalent un-treated cells, **p < 0.05, IFN-γ treated uninfected cells compared with IFN-γ treated infected cells, Fig. 6B, *p < 0.05, actinomycin D treated cells compared with equivalent untreated cells).

IFN-γ had no significant effect on sICAM-1 gene expression in both uninfected and HRV-infected NHBE cells (Fig 5B), suggesting that sICAM-1 release may be regulated at a transcriptional level. Accordingly, separate experiments investigated effects of cycloheximide and actinomycin D on sICAM-1 release. Irrespective of the presence of infection, cycloheximide inhibited sICAM-1 release from IFN-γ stimulated uninfected and infected NHBE cells at 24 h (*p < 0.05, Fig 6A), whilst producing a significant reduction at 96h (*p < 0.05, Fig 6A). Trypan blue exclusion assay demonstrated that cycloheximide had no significant effect of cell viability (95 ± 2.1%). Actinomycin D abolished sICAM-1 release only at the 96 h time point (Fig 6B). Actinomycin D did not have a detrimental effect on cell viability (96 ± 1.8%).

### Role of proteolysis in sICAM-1 production

**Figure 7:**
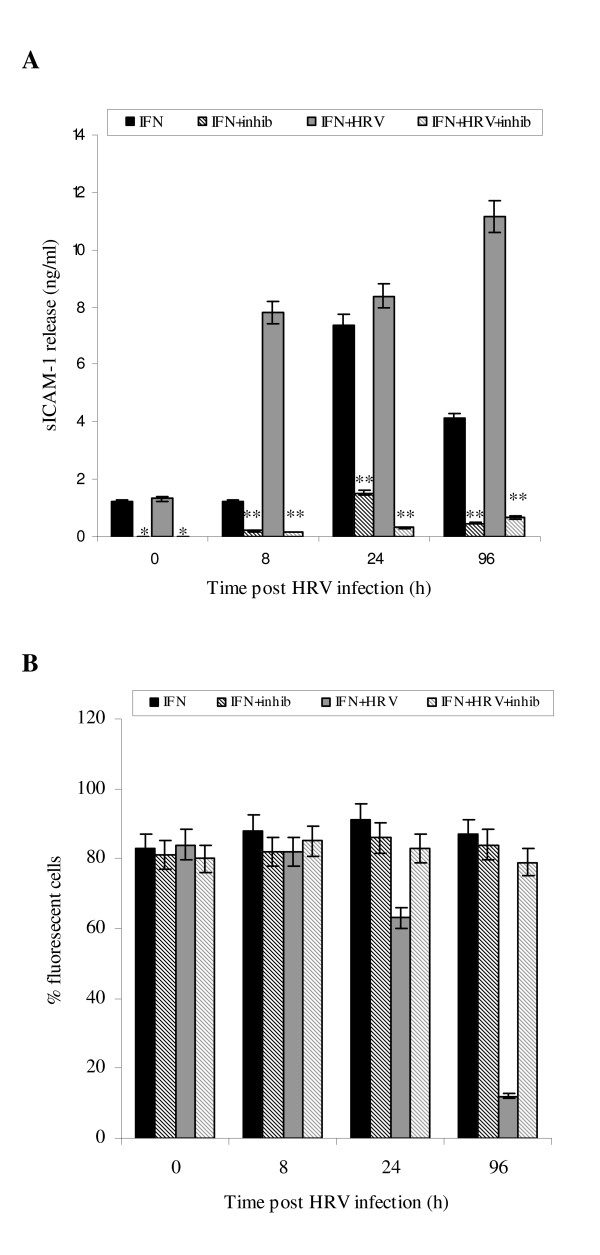
**Effect of protease inhibitors on sICAM-1 release (A) and mICAM-1 levels (B)**. Cell cultures were pre-incubated with 1 complete™ mini tablet/10 ml of media; sICAM-1 in associated supernatants was assayed using ELISA. Data are mean ± S.E. of three separate experiments (Fig. 7A, *p < 0.01, **p < 0.05, IFN-γ treated infected cells pre-incubated with protease inhibitors compared to equivalent untreated IFN-γ treated infected cells). mICAM-1 protein expression was measured using immunofluorescence. Data are mean ± S.E. of three separate experiments (Fig. 7B).

As the sICAM-1 mRNA transcript failed to increase in response to IFN-γ and HRV infection, it followed that ICAM-1 gene splicing was not the primary mechanism underlying sICAM-1 release in equivalent conditions. Equally, observed soluble receptor secretion was associated with simultaneous down-regulation of the mICAM-1 form in IFN-γ-biased NHBE cells during HRV-14 infection; the present authors postulated that sICAM-1 release under these conditions could likely be secondary to proteolytic cleavage of the membrane-bound form. Presence of this mechanism was shown in separate experiments incorporating broad spectrum protease inhibitors which blocked mICAM-1 cleavage, demonstrating a reduction in sICAM-1 release in IFN-γ treated uninfected and HRV-infected cells throughout the experimental time period (Fig 7A *p < 0.01,**p < 0.05, protease treated versus untreated cells); whilst mICAM-1 expression in equivalent IFN-γ treated uninfected and HRV-infected cells remained elevated at all time points (Fig 7B *p < 0.05 protease treated versus untreated cells).

### Consequent effects on viral titres

**Figure 8:**
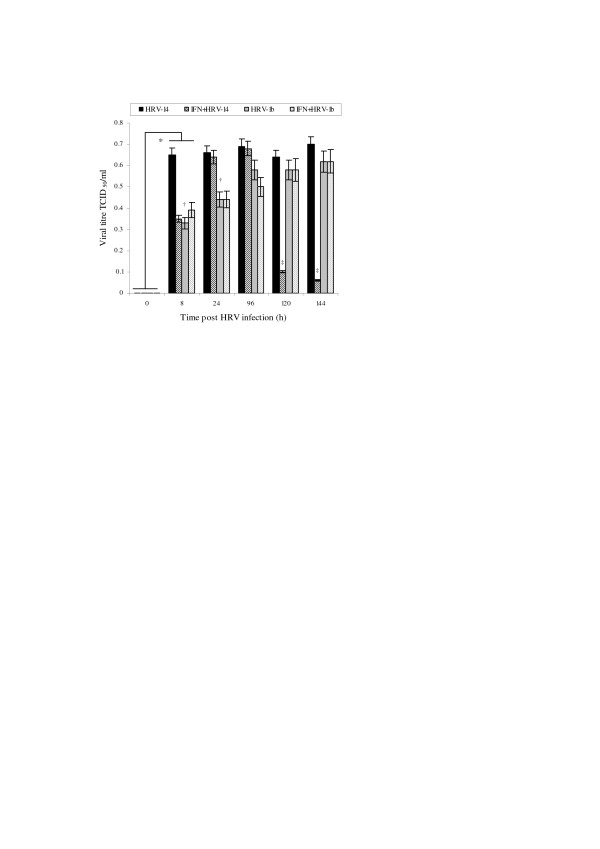
**Effect of IFN-γ cell preconditioning (1 ng/ml for 24 h) on viral titres (0–144 h)**. Viral titres in cell culture supernatants were assayed using TCID_50 _method. Data are mean ± S.E. of three separate experiments (Fig. 8, *p < 0.05, HRV-infected cells at 0 h compared HRV-infected cells at 8 h, ^‡^p < 0.05, IFN-γ treated HRV-14 infected cells compared to un-treated HRV-14 infected cells and IFN-γ treated HRV-1b infected cells and untreated HRV-1b infected cells).

To relate above observations on mICAM: sICAM-1 isoform differential processing in the context of IFN-γ effects and consequence on cell infectivity, viral titres were performed on appropriate test cultures. A steep increase in viral titres was observed in untreated HRV-infected NHBE cells at 8 h, remaining elevated thereafter. In contrast, IFN-γ preconditioning of cells followed by HRV-14 infection induced a down-regulation in viral titres at 8 h compared to untreated infected cells and HRV-1b infected cells (*p = 0.05, Fig. 8). At 24 and 96 h, there were no significant differences in recovered viral titres from infected cell cultures irrespective of IFN-γ pre-treatment. Significantly, viral titres from HRV-1b infected cells were less than those observed from HRV-14 infected cells at 8 and 24 h (^†^p < 0.05 compared to HRV-14 infected cells). To investigate whether local changes in the mICAM-1: sICAM-1 levels could influence epithelial cell infectivity, experiments were extended to examine viral titres at 120 and 144 h. Interestingly, notable differences were observed at these later time points; specifically viral titres from IFN-γ treated HRV-14 infected NHBE cells were greatly reduced compared to untreated HRV-infected NHBE cells and untreated and treated HRV-1b infected cells (^‡^p < 0.05, Fig. 8).

All the above experiments were repeated using NHBE cells from two further sources; apart from differences in response magnitude, the pattern of data was similar.

## Discussion

The present authors describe for the first time that IFN-γ can exert a simultaneous differential effect on both forms of the rhinovirus receptor, ICAM-1, in target infected cells. Specifically, the membranous form of ICAM-1 is down-regulated in IFN-γ-biased epithelial cells exposed to the major group rhinovirus, HRV-14, whilst sICAM-1 release is enhanced.

The current experiments demonstrate that mICAM-1 expression is induced by both major and minor group rhinoviruses. Since both group viruses utilise distinct cellular receptors [21], the observed induction in mICAM-1 expression appears to be not receptor restricted. However, when NHBE cells are first pre-conditioned with IFN-γ and subsequently infected, seminal different response patterns are observed between major and minor group rhinoviruses. Expression of mICAM-1 is down-regulated within 24 h in IFN-γ-biased HRV-14-infected cells, whilst mICAM-1 levels remain elevated in equivalent mediator conditioned cells infected with the minor group serotype. It follows that the effects of IFN-γ and major group rhinovirus together are likely to be receptor mediated. These data support previous findings by Sethi *et al *(1997) who demonstrated a reduction in mICAM-1 expression in response to IFN-γ and HRV-14 in separate cell lines. Whilst these observations require to be confirmed *in vivo*, there are potential implications for the capability of IFN-γ to modulate selectively HRV-14 cell receptor expression, particularly as mICAM-1 is critical in both inflammatory cell mobilisation and virus-host cell binding/internalisation. A down-regulation in mICAM-1 expression on the epithelial cell surface may limit rhinovirus infection by decreasing the number of receptors available for virus binding; whilst possibly also reducing the magnitude of subsequent effector response at the target site.

The current study next explored possible molecular mechanisms by which mICAM-1 down-regulation may occur in IFN-γ-biased cells exposed to HRV-14. As IFN-γ-mediated effects were observed to be sensitive, albeit not wholly, to pharmacological inhibition with cycloheximide and actinomycin D, the authors postulated that modulation of ICAM-1 expression could at least be facilitated at the transcriptional and translational level. There is abundant evidence that STAT-1 signalling is a key regulatory pathway; although not necessarily mediating all effects of IFN-γ [18], it made sense to first determine whether STAT-1 regulation is involved in mICAM-1 modulation during HRV-14 infection. The present data show that IFN-γ induces phosphorylation of STAT-1; its effects on membranous ICAM-1 levels are inhibited in a dose-dependent fashion by AG490 in the absence of infection. These data suggest that the selective transcriptional actions of IFN-γ are likely to be mediated by a conventional JAK/STAT signal transduction pathway, whether wholly or partly in uninfected cells. Interestingly the present authors did not observe AG490 inhibition on mICAM-1 protein levels in HRV-infected cells. This may be due to the fact that this signalling pathway is suppressed by HRV in infected cells. Moreover, the magnitude of STAT-1 phosphorylation was lower after 30 mins, though not after 15 mins, treatment in HRV-infected cells compared with uninfected equivalents. The authors are aware of several reports on virus interference with IFN-γ signalling. Respiratory viruses such as Sendai, Cytomegalovirus, Adenovirus, Vaccinia, simian virus 5, have been shown to reduce STAT phosphorylation [22-26]. It is possible that human rhinoviruses may behave similarly. In addition, HRV proteins may interfere directly with host JAK/STAT signalling mechanism preventing interferon-induced transcriptional responses. Several viruses have been demonstrated to employ this mechanism [27-29]. It follows that STAT-1 phosphorylation may be inhibited by HRV without affecting other molecules such as JAK involved in the signalling cascade. However, whilst HRV-14 manipulation of interferon-dependent immunity is possible, further exploration of the interaction between HRV-14/IFN-γ/JAK/STAT signalling is required to explain the observed lack of AG490 inhibition on mICAM-1 levels in HRV-infected cells; equally other signalling molecules may be operative.

The simultaneous effects of IFN-γ and rhinovirus infection were also explored on the soluble form of ICAM receptor. IFNγ induces a small but significant increase at 8–96 h in sICAM-1 release from uninfected cells. In the presence of major group rhinovirus, sICAM-1 release from IFN-γ-biased cells is augmented in a time-dependent manner. In contrast, infection with the minor group rhinovirus had no significant effect on sICAM-1 release. The concurrent enhanced sICAM-1 release and mICAM-1 downregulation during HRV-14 infection could play a pivotal function in limiting HRV-14 infection within a biased IFN-γ milieu. The present authors postulate that this differential ICAM-1 isoform processing may create an anti-viral influence by reducing the density of the HRV docking target on the cell surface, whilst the released soluble ICAM-1 molecules serve as a decoy to prevent further HRV binding to epithelial cells and infection by binding to virus particles.

Whilst HRV replication was not measured directly, the present data demonstrate that changes in relative expression levels of membrane-bound and secreted ICAM-1 forms are followed by reduction in analysed HRV-14 titres. Viral levels were reduced in IFN-γ treated HRV-14 infected cells at 8 h compared to untreated HRV-14 infected cells. It is possible that the observed reduction in viral titres at this time point reflects a direct effect of IFN- γ on viral replication, although this is just hypothesis at the moment. In addition, at 120 and 144 h post infection, epithelial cell infectivity appeared to be reduced in IFN-γ treated HRV-14 infected cells compared to untreated cells infected with HRV-14 and both IFN-γ treated and untreated HRV-1b infected cells. These observations are consistent with previous studies which also demonstrated reduced viral titres in IFN-γ treated and HRV-14 infected H292 cells [14]. Since these observations appear to be specific to the major group rhinovirus that utilise ICAM-1 as its cellular receptor, the decrease in viral titres are likely to be a consequence of IFN-γ on differential modulation of the ICAM-1 variants. Whilst it is possible that the observed later effects on viral titres may be secondary to *de novo *IFN-γ production inhibiting viral replication, in our previous studies we did not observe increasing IFN-γ levels over the experimental period. The relevance of defense mechanisms driven by IFN-γ-induced processing of the HRV-14 receptor for host epithelial cell infectivity remains to be clarified in future studies.

The current investigation conducted preliminary experiments to explain possible source/s for the enhanced soluble isoforms. The underlying mechanisms responsible for IFN-γ induced modulation of sICAM-1 levels during HRV infection include alternative splicing of the ICAM-1 gene and proteolytic cleavage of the membranous form. Alternative splice donor site selection results in a reading frameshift of the ICAM-1 gene with elimination of the transmembrane and cytoplasmic domains [19]. However, the present authors concluded that gene splicing is not the predominant mechanism driving sICAM-1 release as mRNA transcript expression is not induced by IFN-γ and HRV-14, suggesting other mechanisms are operative. However, Actinomycin D dramatically reduced sICAM-1 release suggesting that gene transcription is involved in the regulation of ICAM-1 receptors in bronchial epithelial cells, albeit not transcription of the actual ICAM-1 gene; possibly the transcription of a gene regulating proteolysis such as MMPs or inhibitors of these proteases. Indeed, separate experiments showed proteolytic cleavage of mICAM-1 as potentially pivotal to sICAM-1 release. Certainly, IFN-γ and HRV-14 together appear to induce an increase in sICAM-1 release, whilst decreasing mICAM-1 expression on the epithelial cell surface. Protease inhibitors with a broad spectrum of activity had no effect on mICAM-1 expression in IFN-γ-stimulated uninfected NHBE cells. However, there was near-complete abrogation of sICAM-1 release from both IFN-γ uninfected and HRV-infected cells incubated with same protease inhibitors. It is thus plausible that during HRV infection, IFN-γ promotes the enzymatic cleavage of mICAM-1 resulting in a downregulation of cell surface mICAM-1, with simultaneous enhanced release of sICAM-1 into the local extracellular environment. Furthermore, the involvement and regulation of selective proteolytic enzymes such as MMPs and caspases in the cleavage of mICAM-1 should also be considered and addressed in future studies, especially in the context of human airway HRV infection. Indeed, keratinocyte and astrocyte models have demonstrated that the sICAM-1 release mechanism is sensitive to metalloproteinase (MMP) inhibitors [9,30] suggesting a critical role for MMPs in the production of sICAM-1. Interestingly, in addition to establishing the role of proteases in the production of sICAM-1, the authors demonstrated the JAK/STAT signalling pathway as a potential mechanism in the regulation of ICAM-1 receptors. The cellular response to virus infection is complex and is likely to involve multiple genes, therefore as the JAK/STAT pathway is a generic signalling mechanism, it may be implicated in the activation of accessory genes, such as proteases/protease inhibitors involved in the regulation of ICAM-1 expression as opposed to the ICAM-1 gene itself.

In conclusion, this current study highlights an interesting modulation of ICAM-1 receptor isoforms by the Th-1 mediator, IFN-γ in the presence of HRV-14 infection. Whilst some findings are preliminary, the present *in vitro *cell model observations are in line with previous data from human experimental rhinovirus studies. Winther *et al*, 2002 [31], demonstrated that nasal epithelial cell mICAM-1 expression is upregulated within 24 h of HRV inoculation, decreasing rapidly to baseline by day 9; with simultaneous increase in sICAM-1 release into nasal lavage, albeit that significance was only noted on day 3. Separate studies by Hildebrandt *et al *(1996) [32] also detected enhanced soluble receptor levels during the acute phase of naturally acquired upper respiratory tract infections. These observations need to be pooled together to design future experiments to determine the relevance of differential ICAM-1 processing on natural rhinovirus infection; this could unravel key molecular targets for anti-HRV pharmacological engineering.

## Competing interests

The authors declare that they have no competing interests.

## Authors' contributions

SCW performed the all experiments and data analysis. MAS is the principal investigator and supervisor of the project, had the original idea and designed the study, and was responsible for obtaining funding. All authors contributed to the write-up and review of the manuscript.
